# Early prognosis of respiratory virus shedding in humans

**DOI:** 10.1038/s41598-021-95293-z

**Published:** 2021-08-25

**Authors:** M. Aminian, T. Ghosh, A. Peterson, A. L. Rasmussen, S. Stiverson, K. Sharma, M. Kirby

**Affiliations:** 1 Department of Mathematics, Colorado State University, Fort Collins, CO 80524 USA; 2 Department of Computer Science, Colorado State University, Fort Collins, CO 80524 USA; 3 Department of Mathematics and Statistics, California State Polytechnic University, Pomona, CA USA; 4 Vaccine and Infectious Disease Organization-International Vaccine Centre (VIDO-InterVac), University of Saskatchewan, Saskatoon, SK Canada; 5 Center for Global Health Science and Security, Georgetown University Medical Center, Washington, DC USA

**Keywords:** Mathematics and computing, Prognostic markers

## Abstract

This paper addresses the development of predictive models for distinguishing pre-symptomatic infections from uninfected individuals. Our machine learning experiments are conducted on publicly available challenge studies that collected whole-blood transcriptomics data from individuals infected with HRV, RSV, H1N1, and H3N2. We address the problem of identifying discriminatory biomarkers between controls and eventual shedders in the first 32 h post-infection. Our exploratory analysis shows that the most discriminatory biomarkers exhibit a strong dependence on time over the course of the human response to infection. We visualize the feature sets to provide evidence of the rapid evolution of the gene expression profiles. To quantify this observation, we partition the data in the first 32 h into four equal time windows of 8 h each and identify all discriminatory biomarkers using sparsity-promoting classifiers and Iterated Feature Removal. We then perform a comparative machine learning classification analysis using linear support vector machines, artificial neural networks and Centroid-Encoder. We present a range of experiments on different groupings of the diseases to demonstrate the robustness of the resulting models.

## Introduction

Transmission routes of human respiratory virus infections are typically via respiratory droplets that arise as a consequence of speaking, sneezing, and coughing. Such infections include a broad range of pathogens including influenza virus, human rhinovirus (HRV), respiratory syncytial virus (RSV), severe acute respiratory syndrome coronavirus (SARS-CoV), Middle East respiratory syndrome coronavirus (MERS-CoV) and the novel coronavirus SARS-CoV-2. These transmission mechanisms are exacerbated by the fact that infected subjects may shed a virus even before the onset of symptoms^1^; the fraction of influenza virus infections that are caused by asymptomatic shedders is estimated to be 10–30%^2^. It is also widely speculated that pre-symptomatic shedding is an important feature of the transmission of COVID-19^3^.

The ability for asymptomatic individuals to shed virus has increased significance given the observation that some of these shedders are responsible for infecting large populations. There is significant evidence that, at least in some cases, the spread of infectious disease can be traced to a small fraction of the population who are typically asymptomatic and shed high volumes of pathogen^4,5^. These individuals are capable of infecting large numbers of the population and, therefore, are referred to as “super-shedders” of a pathogen, or “super-spreaders” of disease. A number of examples of this phenomenon have been documented including typhoid, tuberculosis, and measles virus^6^. There is evidence that super-shedders play a pivotal role in the spread of disease such as in the SARS outbreak of 2002^7^. There is also emerging evidence of super-shedders in the COVID-19 pandemic^8–10^. However, little is known about the specific host responses to infection that contribute to shedding.

The primary aim of this investigation is the development of a predictive model capable of distinguishing pre-symptomatic infected individuals from uninfected controls. This is accomplished via the analysis of host gene expression profiles of blood and the exploration of signatures of shedding before symptom onset. Based on evidence from machine learning analytics, these sample measurements provide significant discriminatory information related to the host immune response to infection soon after infection and significantly before the development of symptoms. Experimental findings suggest that gene expression associated with the immune response changes significantly, even within the first 8 h after infection. As such, they provide a wealth of quantitative information that can to be decoded to reflect discriminative signatures that can be used for predictive models as well as biological discovery.

To accomplish our predictive modeling aim we focus on identification of signatures that are predictive of shedding within the first 32 h post-exposure. We begin with a visual exploration of data from infected individuals and visually demonstrate how clearly the movement of host response to disease through time is and lend visual justification to our premise that the prognosis, i.e., the prediction of the course of a disease, can be determined in the first 32 h. We provide visualizations to support this hypothesis. Next we implement feature selection algorithms designed to extract discriminatory sets on a variety of dataset groups and time windows. These machine learning algorithms, provide evidence that these feature sets are capable of identifying shedders in the first 32 h after exposure. Lastly, we show that these predictive features reflect biologically relevant host responses that may contribute directly to shedding.

## Results

### Method overview

We analyze microarray data of gene expression profiles of blood samples from individuals at different time points who were infected with HRV, RSV, H1N1 and H3N2 as part of several clinical challenge studies^11^. This data is publically available on the NCBI Gene Expression Omnibus (GEO) database with identifier GSE73072. The data was normalized using standard RMA (Robust Multi-array Average) normalization procedure on the entire dataset^12^. We implemented additional strategies for removing batch effects using Limma (LInear Models for MicroArray) including Subject ID and Study ID normalization^13^. The features of the datasets are probe set identifications associated with gene expression.

The machine learning experiments were performed using binary classes. We take the negative class $C^-$ as the class of controls, i.e., samples prior to infection, associated with the studies selected for the model. Our positive $C^+$ class is comprised exclusively of samples from pre-symptomatic shedders. These samples were collected in the first 32 h after inoculation and prior to actual shedding. All samples in the positive class are from subjects who eventually will test positive for the challenge virus as confirmed by nasal swab tests.

A novel tactic employed here is to separate the positive classes $C^+$ as a function of time. Thus, we partition the positive samples into four windows of 8 h over the first 32 h. Again, although the samples in the positive class are from subjects who are not yet symptomatic, or shedding virus, they will *all* at some point in time be symptomatic and shed, i.e., will become clinically positive.

Our analysis begins by using machine learning algorithms to first identify *all* features capable of discriminating between the control class $C^-$ and the $C^+$ positive shedder class for four distinct 8 h time bins spanning hours 1–8, 9–16, 17–24 and 25–32. Table 1 shows the number of samples for the control class and positive class for the different studies and time bins that are used to find the discriminatory feature sets. The set of discriminatory features for the first 8 h are found independently from the set of features identified in hours 9–16, and so on. These features are then ordered according to their estimated predictive power. Once ordered sets of discriminatory features have been established for each experiment, we conduct a Leave-One-Subject-Out (LOSO) cross validation experiment to evaluate the features.

The net result of the feature extraction described above was Feature Sets 1–3, each constructed for the 8 h time intervals over the first 32 h. Feature Set 1 uses only the influenza data in the iterative feature removal (IFR)^14^ applied to each time-bin separately; Feature Set 2 is constructed similarly on the combined HRV, RSV, H1N1 and H3N2/DEE5 data while Feature Set 3 employs H1N1 (both sets) H3N2/DEE5 and HRV (both sets). This seemingly elaborate partitioning was done to explore the ability of different viruses to produce generally discriminatory features. The resulting feature sets are used to perform the machine learning experiments in this paper.

Given that the number of control samples is generally a factor of two or more larger than the number of positive samples in $C^+$, we compute classification accuracy using a Balanced Success Rate (BSR). BSR is defined as the average of the true positive rate and the true negative rate for a binary classification problem and accounts for imbalances between the classes.

**Table 1:** Number of samples by study and time bin interval in hours over the first 32 h.

Disease/study	Controls	Shedders 1–8	Shedders 9–16	Shedders 17–24	Shedders 24–32
HRV/Duke	53	32	34	34	16
HRV/Uva	40	22	24	24	12
RSV/DEE1	40	13	13	13	13
H3N2/DEE2	34	11	11	11	11
H1N1/DEE3	46	13	13	13	13
H1N1/DEE4	38	15	15	15	12
H3N2/DEE5	21	10	10	10	10

### Exploratory visualizations

There is significant experimental evidence that the immune response may be viewed as a sequence of biological manufacturing processes. There is a collection of interacting biological pathways that produce a temporally evolving series of molecular defense mechanisms in an organized cascade. As such, the genes being expressed in these pathways at any given time after the initial infection will be changing as a reflection of this temporal progression of the host response. Understanding the temporal evolution of the gene expression is potentially important for accurate diagnosis and prognosis of patient outcome.

**Figure 1:**
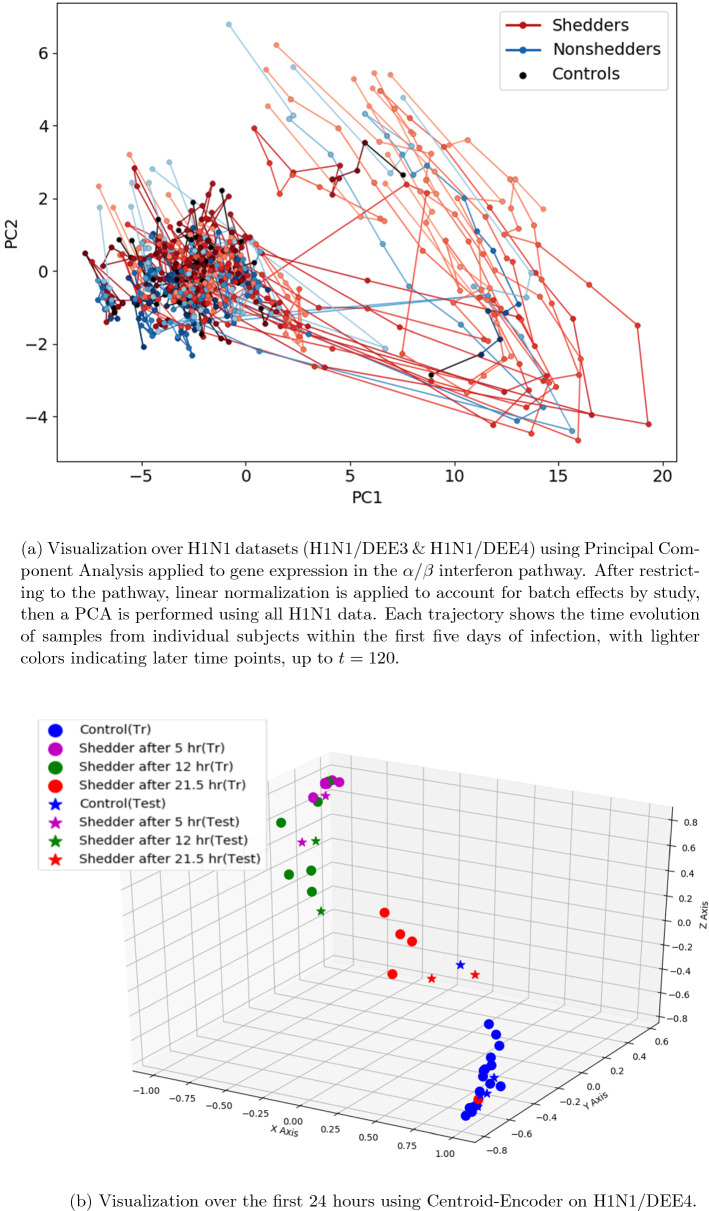
Visualizations of the $\alpha$-$\beta$ interferon signaling pathway on H1N1 datasets.

As an illustration of the temporal evolution of the immune response, the host gene expression of the Reactome $\alpha$/$\beta$ interferon pathway is visualized in Fig. 1. The $\alpha$/$\beta$ interferon pathway plays a crucial role in host responses to viral infection such as the regulation of Type I interferon responses^15^. A visualization of this pathway illustrates the evolution of the host immune response to H1N1 as seen in Fig. 1. Genes associated with this pathway were extracted from pathway databases available via the Molecular Signatures Database^16,17^. Association of Entrez Gene IDs to microarray probe identifiers was done using the associated microarray platform file, resulting in 94 probes of interest.

A standard principal component analysis (PCA)^18^ visualization of this gene expression data for all subjects and all time points is shown in Fig. 1a. The 94 probes associated to the pathway for each sample are projected to the 2 directions with maximum variance after mean subtraction. The reduced gene expression data associated with the H1N1 infection (studies DEE3 and DEE4) for this pathway are seen to remain relatively constant for subjects who do not shed virus. The non-shedder trajectories are shown in the blue. In contrast, subjects who are shedders have an excursion from the health state, seen as trajectories in red in Fig. 1a. The fact that these values return to their nominal value after some time indicates that biomarkers associated with this pathway are not uniformly discriminatory over the time evolution of the disease. This underlines the time-dependent nature of gene expression biomarkers for predictive modeling of the host immune response using machine learning.

A neural network dimension reduction technique is applied to the $\alpha$–$\beta$ interferon signalling pathway for the H1N1/DEE4 data shown in Fig. 1b; we select a single data set to minimize variation due to batch effects. The visualization is achieved using the nonlinear Centroid-Encoder (CE), a recently proposed supervised variation on the nonlinear autoencoder, i.e., CE visualization exploits class labels^19,20^. In this example the class labels correspond to the time intervals of the $\alpha$–$\beta$ interferon signalling pathway. The nonlinear and supervised aspect of this reduction process make it ideal for extracting early variations in the pathway evolution. In Fig. 1b, the trajectories of the shedders exhibit large excursions (to purple) already *after 5 h*. The trajectories for this pathway appear to return towards the nominal state over the course of 24 h. The evolution of the location of the data neighborhoods serves as a geometric characterization of the biological processes. In contrast to PCA, the visualization characterizes the significant temporal variation in the first 24 h after exposure to the H1N1 pathogen.

**Figure 2:**
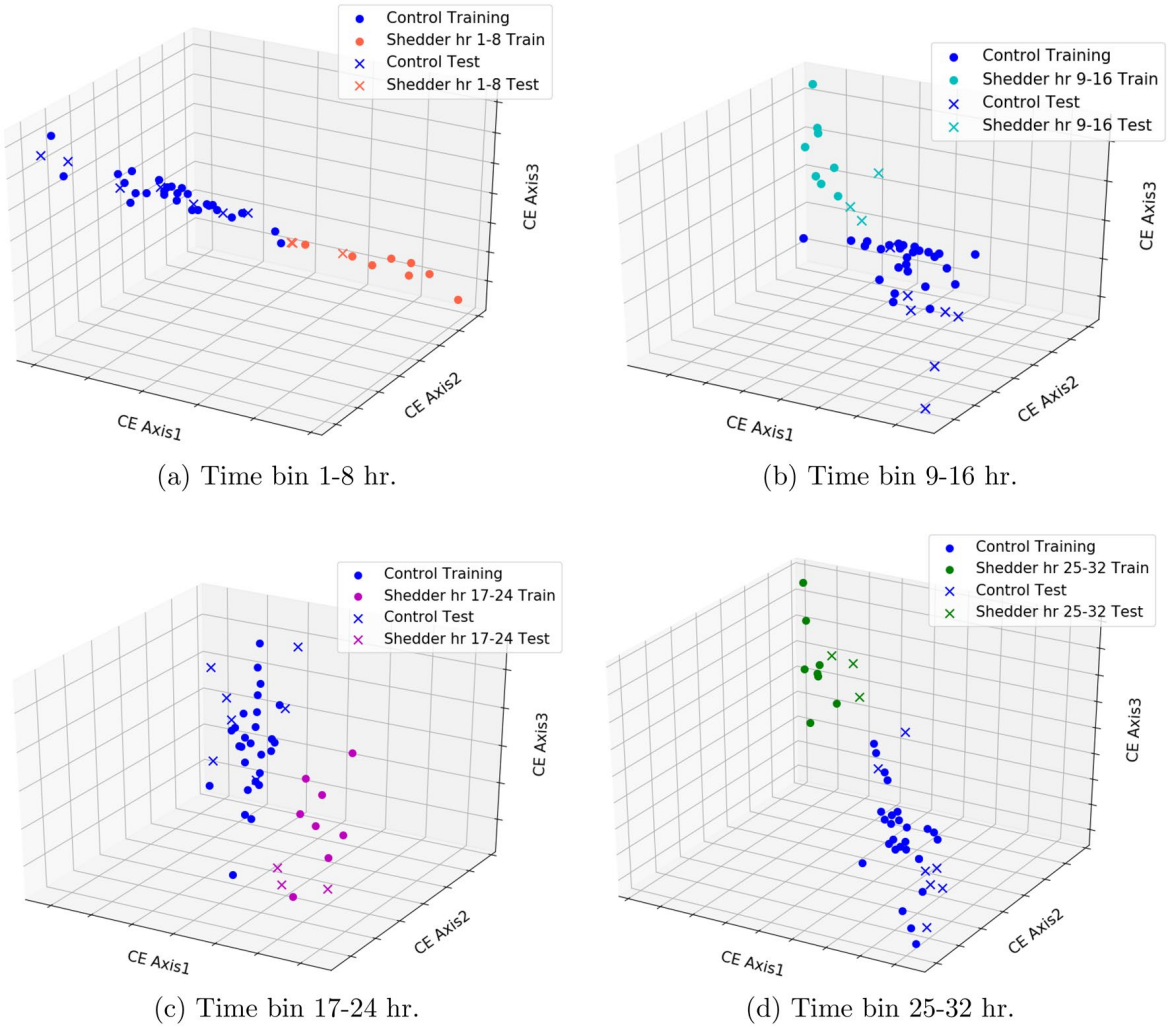
Three dimensional visualization of control and shedders using Centroid-Encoder on the H3N2 study (GSE73072/DEE2) which is not used to extract the features. The features are from Feature Set 3. A total of $80\%$ of samples from each class is used to train the model and rest of the $20\%$ is used as test data.

Another look at this early temporal evolution is shown for Fig. 2 that visualizes the H3N2/DEE2 dataset in the first 24 h after infection using CE on optimized features. Specifically, in this figure we explore the 3D visualization of the controls versus the shedders using Feature Set 3 partition by time interval. In particular, in Fig. 2 the gene expression values of the subjects in Feature Set 3 are shown for the given time interval where their discriminatory capability is at a maximum. This is in contrast to the pathway analysis above were the features were fixed while their expression varied over time.

**Figure 3:**
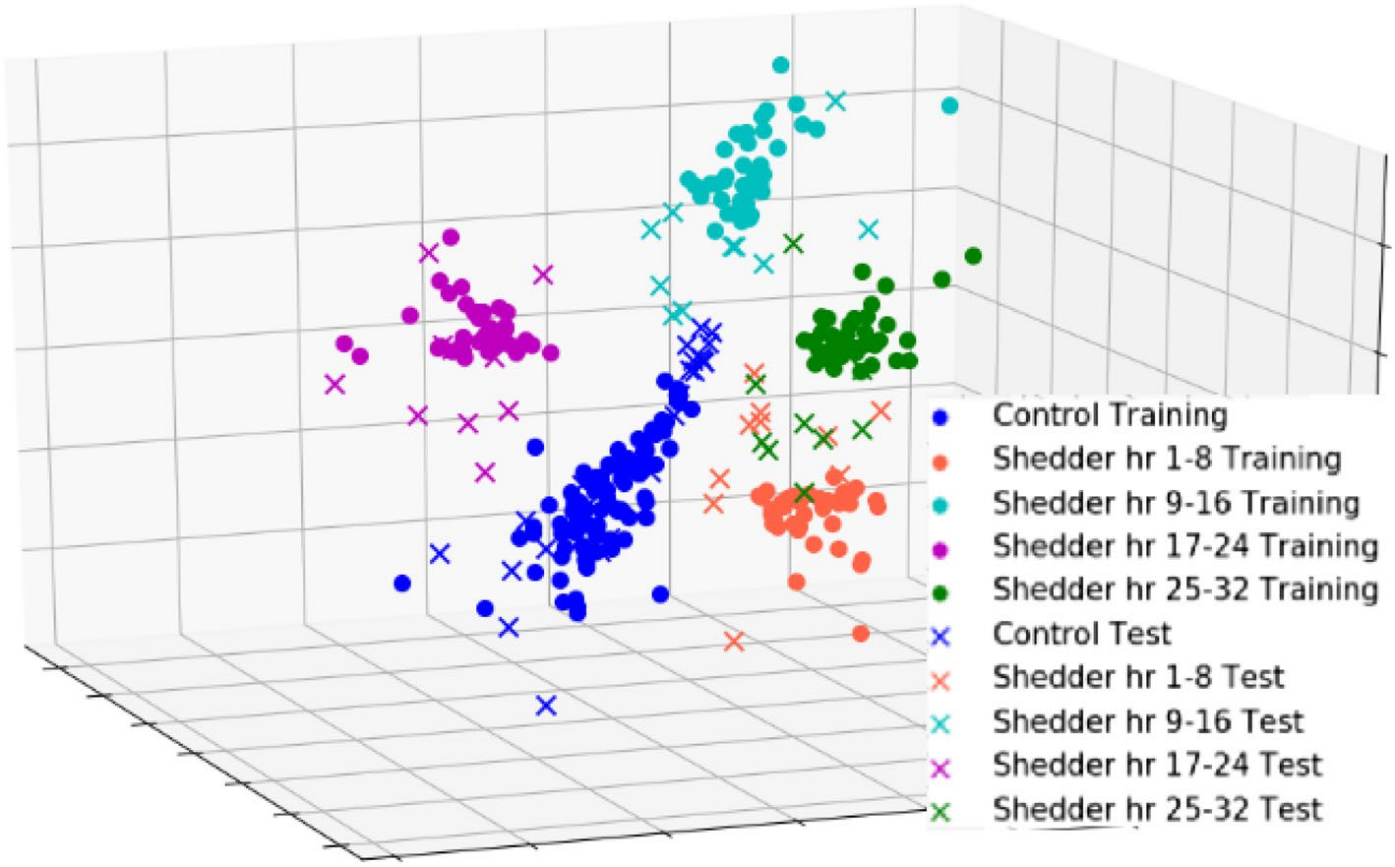
Pooled data visualization using the union of top features identified as discriminatory in the first 32 h. Blue represents controls, orange is time 1–8 h, cyan is time 9–16 h, magenta 17-24, green is 25-32. Training data are circles and test data are crosses. This is retrospective data with Subject ID normalization.

In our final visualization, Fig. 3 displays the entire Feature Set 1 pooled across the four time bins. All the data in the first 32 h from the four H1N1 and H3N2 data sets are mapped to three dimensions using Centroid-Encoder with this pooled feature set. This visualization provides additional validation that our representation has captured discriminatory information related to early infection. Classification rates using these pooled features are discussed in what follows.

In summary, this suite of visualizations consistently suggests that there is signficant temporal evolution in gene expression that could permit the classification of shedders in the first 32 h. As a group, they provide a compelling body of evidence to suggest that the biological response to infection is extremely rapid and as such provide visual support for the successful prognosis experiments that follow. In other words, the fact that these low-dimensional visualizations provide clear delineation of the change in gene expression over time serves to validate the machine learning classification results we will present in what follows.

### Prognosis first 32 h

**Table 2:** Analysis of the influenza data using Feature Set 1 with limma normalization on study ID.

Bin	No. features	Linear SVM	Centroid-Encoder	ANN
1–8	136	$99.00 \pm 0.00$	$99.18 \pm 0.55$	$98.13 \pm 0.58$
9–16	38	$100.00 \pm 0.00$	$98.14 \pm 0.61$	$98.25 \pm 0.59$
17–24	200	$96.00 \pm 0.00$	$94.51 \pm 1.25$	$94.21 \pm 1.29$
25–32	60	$100.00 \pm 0.00$	$96.31 \pm 0.98$	$96.89 \pm 0.68$

**Table 3:** Balanced success rate (BSR) of LOSO testing on three test studies using Feature Set 1.

Time bin	Classifier	Test data set		
		HRV/Duke	HRV/UVa	RSV/DEE1
1–8	Linear SVM	$81.54 \pm 0.00$	$80.34 \pm 0.00$	$89.81 \pm 0.00$
	Centroid-Encoder	$85.79 \pm 1.30$	$94.81 \pm 1.82$	$88.92 \pm 1.82$
	ANN	$85.51 \pm 1.58$	$93.90 \pm 1.01$	$92.72 \pm 1.51$
9–16	Linear SVM	$90.76 \pm 0.00$	$87.92 \pm 0.00$	$80.87 \pm 0.00$
	Centroid-Encoder	$91.14 \pm 1.20$	$94.14 \pm 0.93$	$89.53 \pm 3.31$
	ANN	$90.49 \pm 2.26$	$94.42 \pm 2.25$	$90.16 \pm 3.00$
17–24	Linear SVM	$73.75 \pm 0.00$	$93.75 \pm 0.00$	$83.37 \pm 0.00$
	Centroid-Encoder	$82.40 \pm 2.26$	$89.36 \pm 1.59$	$88.89 \pm 1.80$
	ANN	$79.94 \pm 2.20$	$89.14 \pm 1.19$	$93.64 \pm 1.54$
25–32	Linear SVM	$83.43 \pm 0.00$	$95.83 \pm 0.00$	$77.02 \pm 0.00$
	Centroid-Encoder	$83.41 \pm 0.91$	$88.94 \pm 2.13$	$59.89 \pm 2.74$
	ANN	$86.41 \pm 1.92$	$88.28 \pm 2.33$	$58.72 \pm 3.27$

Data is normalized using Limma on Subject ID.

**Table 4:** Balanced success rate (BSR) of LOSO testing on three test studies using feature set 1.

Time bin	Classifier	Study	
		HRV/(Duke and UVa)	HRV/(Duke a UVa) and RSV/DEE1
1–8	Linear SVM	$84.74 \pm 0.00$	$82.06 \pm 0.00$
	Centroid-Encoder	$95.02 \pm 1.35$	$82.29 \pm 1.30$
	ANN	$91.70 \pm 1.32$	$84.64 \pm 1.80$
9–16	Linear SVM	$93.21 \pm 0.00$	$90.37 \pm 0.00$
	Centroid-Encoder	$94.03 \pm 2.16$	$91.76 \pm 0.76$
	ANN	$93.93 \pm 1.30$	$91.99 \pm 0.76$
17–24	Linear SVM	$81.58 \pm 0.00$	$78.82 \pm 0.00$
	Centroid-Encoder	$89.72 \pm 1.87$	$83.02 \pm 1.71$
	ANN	$85.42 \pm 1.29$	$85.69 \pm 1.19$
25–32	Linear SVM	$88.21 \pm 0.00$	$85.46 \pm 0.00$
	Centroid-Encoder	$89.89 \pm 2.72$	$82.51 \pm 1.89$
	ANN	$87.44 \pm 1.95$	$83.23 \pm 0.96$

Limma is applied using subject ID to normalize the data.

**Table 5:** Balanced success rate (BSR) of LOSO testing applied on the study H3N2/DEE2 using the features from the other 6 data sets, i.e., Feature Set 2.

Time bin	Classifier	H3N2/DEE2
1–8	Linear SVM	$87.97 \pm 0.00$
	Centroid-Encoder	$93.66 \pm 2.21$
	ANN	$89.27 \pm 2.18$
9–16	Linear SVM	$100.00 \pm 0.00$
	Centroid-Encoder	$98.89 \pm 1.79$
	ANN	$97.83 \pm 1.82$
17–24	Linear SVM	$86.36 \pm 0.00$
	Centroid-Encoder	$78.07 \pm 1.81$
	ANN	$85.17 \pm 2.33$
25–32	Linear SVM	$89.44 \pm 0.00$
	Centroid-Encoder	$93.88 \pm 1.38$
	ANN	$90.55 \pm 4.21$

Limma is applied on Study id.

Here we present the results machine learning algorithms used to determine biological signatures of shedding in the first 32 h. All of the feature selection algorithms in this paper are based on a modification of the iterative feature removal algorithm based on sparse support vector machines as originally described in^14^. The resulting discriminatory features are used to build classifiers to predict whether a sample is a shedder, or not. In “Methods”, we discuss a mechanism for ordering the features by their importance for discrimination.

The first experiment concerns Feature Set 1 which is obtained by applying time bin feature selection to the four influenza data sets. We perform a LOSO classification on the same data, the influenza studies, using these features. Since the discriminatory features were identified on the same data sets that are then predicted in this experiment, we view this analysis as *retrospective.* The results in Table 2 show that the feature sets identified are capable of discriminating controls from shedders with high accuracy. The classification experiments were run using LOSO across three different algorithms including linear support vector machines (SVM), an artificial neural network (ANN) classifier and Centroid-Encoder (CE); each technique is described in “Methods”. The non-convex optimization problems, CE and ANN, were each repeated 15 times. Note that the unique SVM solution produces a single average LOSO error. With BSR classification accuracies in the mid- to high-nineties we conclude that Feature Set 1 captures the signals that distinguish controls from eventual shedders for influenza for each time bin. These feature sets can now be mined for biological mechanisms.

The next task is to see how the feature sets generalize to prospective data sets. With this in mind we apply Feature Set 1, determined on the influenza data, to the problem of prognosis of the respiratory infections RSV and HRV at different time bins. This classification task is again the discrimination of controls from eventual shedders.

Our first test is to use the RSV/DEE1 and HRV/Duke, HRV/UVa data sets individually as prospective datasets; we show the results of this experiment in Table 3. We emphasize that these features were computed on H1N1 and H3N2 data and tested on other respiratory infections. Our visualizations lead us to expect good classification rates in first 8 h time frame and this is indeed the case with nonlinear methods averaging near 90% accuracy. The relatively lower performance for SVM suggests that the boundary separating the controls from the shedders is nonlinear. It appears that HRV and RSV accuracies are comparable suggesting that the classification has more to do with non-pathogen specific aspects of the immune response. The story remains much the same in hours 9–16 but one might infer that the descision boundary for HRV has flattened given the improved performance of the SVM classifier. We see a decrease in the accuracy at 17–24 h for the HRV/Duke dataset. The first significant decline in prediction accuracy is for RSV in the time window 25–32 h. Given the significant biological differences between RSV and influenza, this may now be a pathogen specific response variation.

We now explore the impact of merging these HRV and RSV studies to create larger data sets. As shown in Table 4, we see classification results improve with the merged set of HRV/Duke and HRV/UVa as well as with the merged set of all three studies HRV/Duke, HRV/UVA and RSV/DEE1. The BSR classification accuracy of the HRV/(Duke and UVa) averages 90–95% over the four time bins for the best method CE. In contrast to Table 3, here we do see a more uniform reduction in prognosis rates with the inclusion of RSV/DEE1.

It is also interesting to explore the predictive value of our models. Recall that positive predictive value (PPV) is the probability that subjects classified as $C^+$ are members of this class while the negative predictive value (NPV) is the probability that subjects predicted to be $C^-$ actually are $C^-$. Applying CE to the combined HRV data data we find the negative predicitive values of our model to be highly accurate. In summary, for CE applied to HRV we found:

**Table:** 

Time bin	PPV	NPV
1–8	87.5	96.77
9–16	94.83	97.85
17–24	84.48	95.70
25–32	82.14	100.00

In our final experiment for predicting controls from pre-symptomatic shedders we apply Feature Set 2 to the experiment of predicting H3N2/DEE2 influenza. The dataset H3N2/DEE2 was sequestered from the feature selection, i.e., it is prospective. Feature sets were found using IFR on the data without H3N2/DEE2 with Limma batch effect removal using Study ID applied before feature selection. We then compare the performance of the prediction on H3N2/DEE2 using the features selected. The results from this experiment are shown in Table 5. Given there is only one dataset (H3N2/DEE2) being used in this experiment no Limma normalization was used since it was not necessary to correct for batch effects in the prognosis model. The high accuracy provides evidence that the time-dependent feature sets are able to effectively discriminate between shedding and controls in the first 32 h. While these results are promising, the relatively small data set may be responsible for introducing additional variability in the results, particularly for the 17–24 h window where there are only 11 samples associated with shedders and 34 controls. It is also possible that this is the result of the increased complexity of the immune response once more defense mechanisms have been activated. Samples that are roughly 24 h apart may also be harder to discriminate due to circadian rhythm similarities.

In the next section, we explore the effect of pooling features. In other words, rather than restricting classification to bins of 8 h intervals, we broaden the bins successively until we collect features from all four time bins as a single feature set.

### Time pooled prognosis of contagion

In this section, we explore the effectiveness of pooling the feature sets determined as optimal for individual time-intervals. In doing this we gain additional insight into the changes of the host response at different stages of the infection. Combining features over time will indicate how useful the union of discriminatory features is to the overall prognostic question. In the first 8 h, the classification is computed exactly as above, using features identified as optimal for the time bin 1–8 h. The classification in the second 8 h interval now uses the features identified as optimal in the first 8 h combined with those that were identified as optimal in the second 8 h period, or time bin 9–16. We refer to this combined interval using the starting and ending time of the included features, e.g., the union of features from bin 1–8 and bin 9–16 as referred to as bin 1–16. Proceeding in this fashion, the bin labeled 1–32 includes features that comprise the union of all the features that were identified as optimal in each of the first four 8-h time intervals. Classification results on data selected from these time bin intervals are provided where the features are pooled as described above.

**Figure 4:**
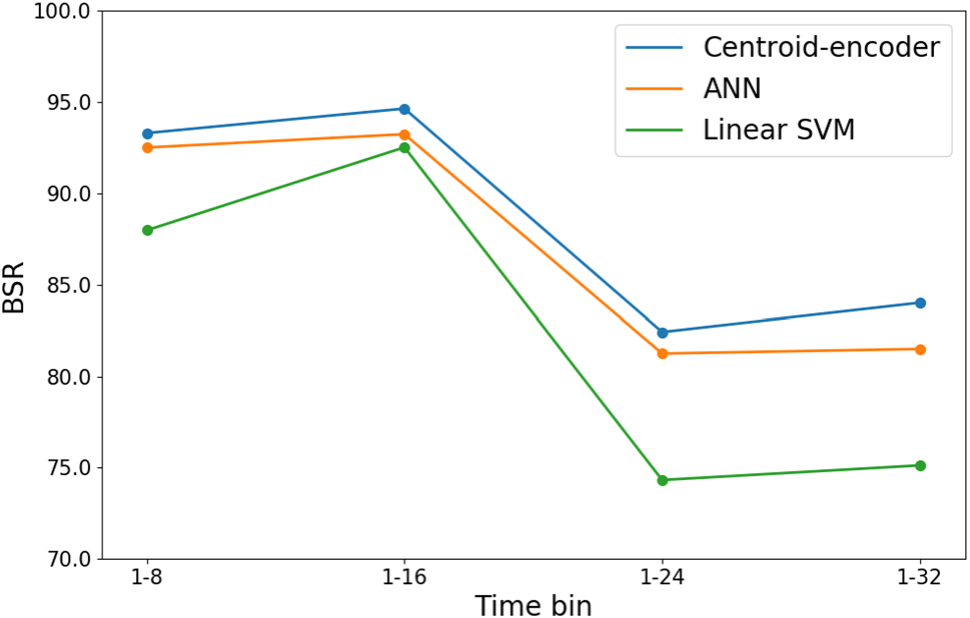
Balanced success rate (BSR) of LOSO testing applied on the study H3N2/DEE2 using Feature Set 3 pooled by windows. Limma normalization is applied on Study ID. There are a total of training samples 264 and 68 test samples.

In this feature pooling experiment we classify the H3N2/DEE2 data set alone using features from the combination of the H1N1, H3N2/DEE5, and HRV, we have omitted RSV given its known dissimilarities with influenza. We refer to the features drawn from these particular studies as Feature Set 3. In Fig. 4, we see the results of applying this pooled approach. A highlight of this graphic is that CE can predict with approximately 84% accuracy that a subject will become a shedder using a pooled set of features from the first 32 h. Perhaps significantly, we conclude that higher accuracies, such as the 95% found when the time bins are known, will require that the machine learning algorithm exploit the temporal evolution of the signatures.

### Temporal evolution of biomarkers

**Figure 5:**
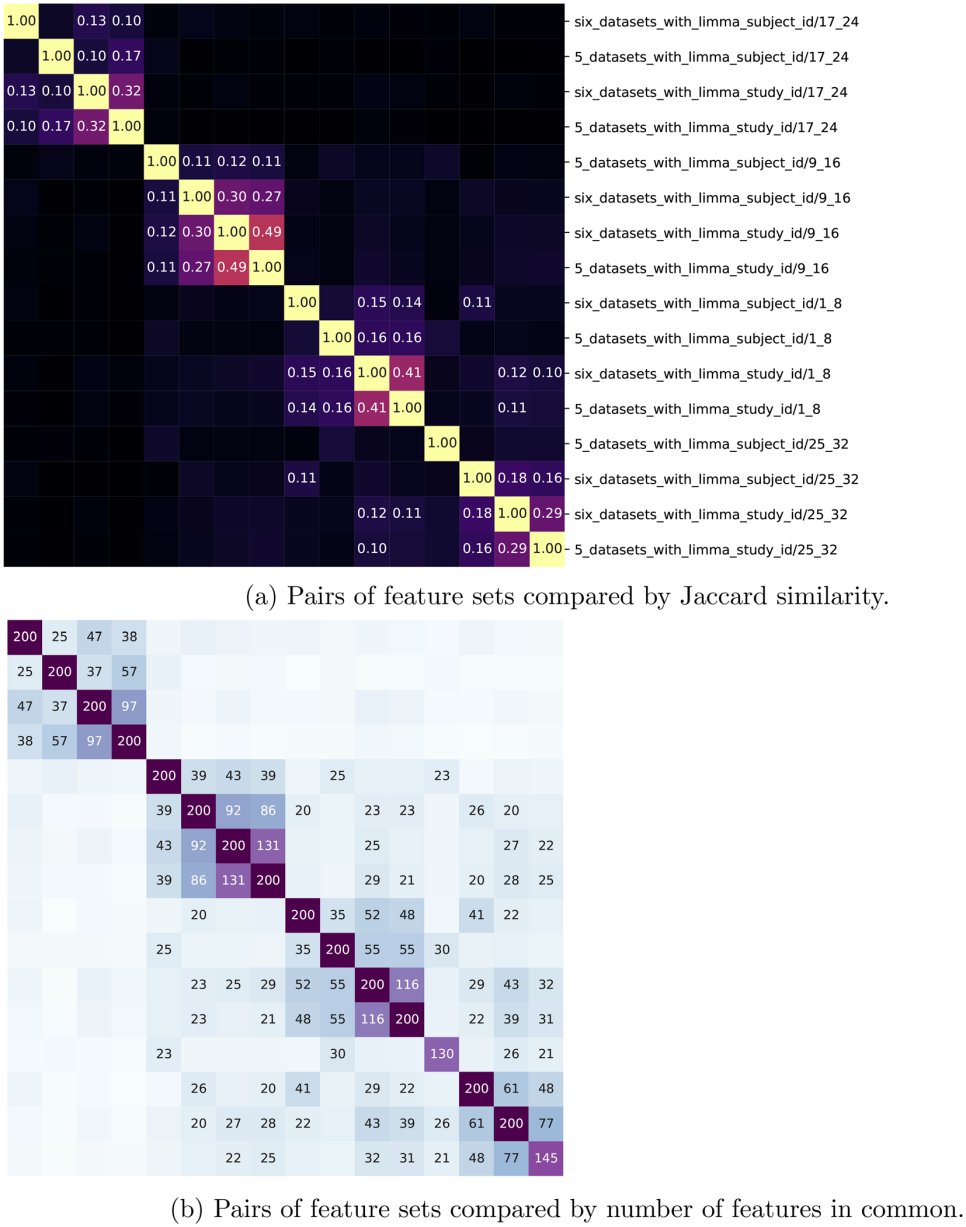
Comparison and clustering of the sixteen distinct feature sets generated based on different subsets of the data. The similarity matrix used for clustering is based on the Jaccard metric between sets measuring the size of the intersection of two sets divided by the size of the union—which ranges from 0 (no features in common) to 1 (perfect match). (**a**) Similarity matrix using the Jaccard metric shown. Two strong clusters and a weaker third cluster is seen primarily based on data inclusion, with variation within-cluster based on preprocessing. Rows follow the format “study_preprocessingtime_range”. For instance, “17_24” refers to hours 17–24 after inoculation. (**b**) The same matrix is shown (same cluster order), but with absolute number of features in common illustrated instead (note most feature sets have 200 features).

In the previous section, we addressed the question of whether biomarkers from different time bins had discriminatory power in other time bins. Here we look at the feature sets themselves and analyze any overlap between features selected for different time bins. In Fig. 5a, we use the Jaccard similarity metric to analyze overlap in Feature Sets 2 and 3 with respect to their different time bins and different limma normalization. In Fig. 5b, we use the same metric and feature sets but the numbers in the figure are the actual number of features in the feature sets.

The Jaccard similarity metric of two sets is the number of elements in the intersection of the two sets divided by the number of elements in the union of the two sets^21^. For example the diagonal in Fig. 5a is 1 since this is the comparison of a set with itself. The only numbers displayed in the top figure are those greater than 0.1 and less than 1. These indicate a small overlap between feature sets. Clusters appear to group based on on time bin for various methods of preprocessing and feature selection, demonstrating some robustness to the process.

Analyzing where these areas of overlap occur, several are different feature selection experiments for the same time bin, indicating, for example, Feature Set 3 (with Limma using study ID) at time bin 1–8 has overlap with Feature Set 2 (with Limma using study ID) at the same time bin 1–8. This is unsurprising since the feature sets were drawn from overlapping data sets, but it is worth further consideration if the differing Limma techniques should have resulted in such low overlap.

The other areas of overlap, which have to do with our analysis of time dependent features, all occur between the first time bin, hours 1–8, and last, hours 25–32. These are minimal but suggest possible circadian rhythm influence or similar cyclical nature of features selected from pre-symptomatic shedders.

Finally we note the lack of overlap between feature sets from time bins 1–8, 9–16, and 17–24. This indicates that the features being drawn are in fact time dependent. Besides not improving classification on different time bins as addressed in the previous section, features drawn in a particular time bin to maximize discrimination between pre-symptomatic shedders and controls have little to no overlap with those in other time bins reinforcing the conclusion that time plays a pivotal role in identifying pre-symptomatic shedders. This validates the feature sets from a modeling prospective moving to the next section we will address the question of whether the feature sets themselves are associated with pathways and genes involved in immune response and viral shedding.

### Viral shedding is linked to suppressed cellular immunity

**Figure 6:**
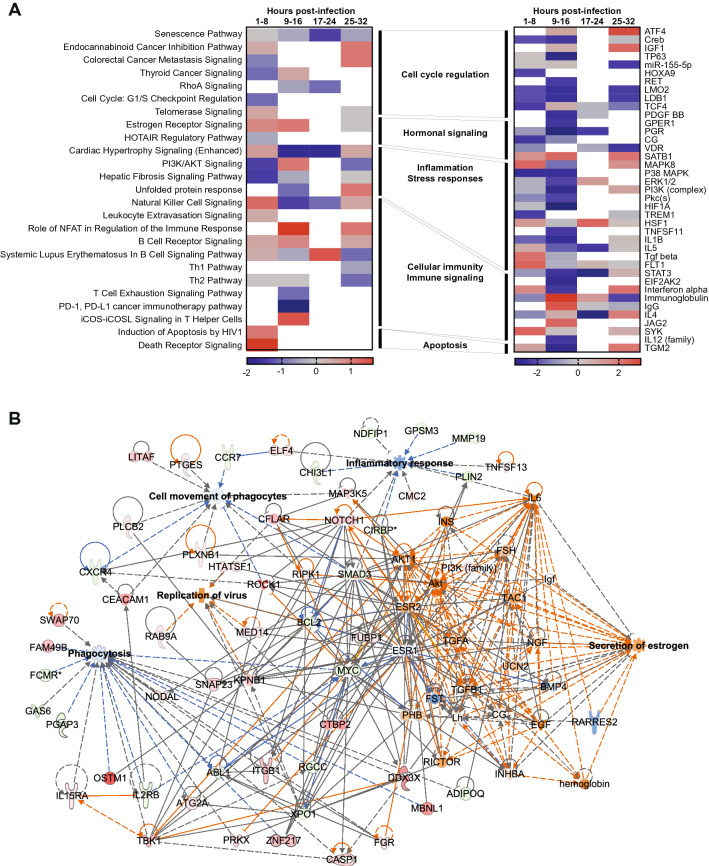
Biological function of molecules associated with virus shedding. (**A**) IPA enriched canonical pathways (left panel) and predicted upstream regulators (right panel) from classifier gene expression in the blood of virus shedders relative to pre-infection controls. Pathways shown had Benjamini–Hochberg-adjusted enrichment p values $< 0.05$ and activation z scores $> |0.5|$. Heatmap shading indicates z score (red: positive z score, predicted activation; blue negative z score, predicted inhibition; gray: significant enrichment, no predicted activation; white: no significant enrichment). (**B**) IPA molecule activity prediction network showing interactions and predicted activity between genes in different functional categories (phagocytosis, cell movement of phagocytes, inflammatory response, and secretion of estrogen). Molecule shading indicates expression (red: upregulated expression relative to controls; green: downregulated expression relative to controls; orange: predicted upregulation; blue: predicted downregulation). Lines indicate interactions (solid: direct interaction; dashed: indirect interaction; orange: predicted activation; blue: predicted inhibition; gray: no prediction).

Now that the validity of features has been assessed through modeling in this section, we assess the validity of the features as biological features associated with viral immune response. To assess the biological significance of the classification signature, we calculated fold gene expression in shedders relative to controls and assessed functional enrichment using Ingenuity Pathway Analysis (IPA) on Feature Set 2. Viral shedding was associated with an early perturbation in canonical pathways associated to cell cycle regulation, as well as suppressed inflammation and stress responses (Fig. 6A). Although B cell activation was sustained over the first 32 h post-infection, other pathways associated with leukocyte migration and cellular immunity were either not enriched or increasingly suppressed. Natural killer (NK) cell activation and pathways linked to T cell differentiation and effector function were inhibited over time, suggesting that cell-mediated immunity in shedders may be delayed or suppressed.

To further investigate the functional implications of these enriched pathways, we used the IPA Upstream Analysis module to identify the predicted activation state of key pathway regulators (Fig. 6B). Predicted upstream regulators demonstrated sustained inhibition of key inflammatory mediators such as mitogen activated protein kinases (MAPK, P38 MAPK, ERK1/2), triggering receptor expressed on myeloid cells 1 (TREM1), phosphatidylinositol 3-kinases/protein kinase B (PI3K/Akt), proinflammatory cytokines such as interleukin (IL)-1$\beta$ and IL-5, and stress factors such as hypoxia-inducible factor-1$\alpha$(HIF1A). Concurrently, cytokines associated with inflammatory suppression such as transforming growth factor $\beta$ (TGFB) were predicted to be activated in the first 8 h after infection. Although type I IFN$\alpha$ was upregulated early, interferon-stimulated genes (ISG; EIF2AK2, STAT3) were inhibited, as were cytokines critical for T cell differentiation (IL-12). Together, functional relationships between genes predicting virus shedding also indicate delayed or suppressed early antiviral responses that allow a productive infection to be established, suggesting a possible mechanism for increased virus replication leading to shedding.

Interestingly, we observed an early upregulation of estrogen receptor signaling in virus shedders. Sex hormones are known to regulate inflammation and have a demonstrable impact on influenza virus infection and pathogenesis^22–26^. Although virus shedding was not specifically associated with sex in these studies, we used the IPA Molecule Activity Prediction analysis module to investigate the relationship between several significantly enriched functional categories in the classifier signature (phagocytosis, phagocyte migration, inflammatory response, and virus replication) and estrogen secretion (Fig. 6). We observed that, phagocytosis, phagocyte migration, and inflammatory responses were predicted to be inhibited, consistent with our findings in both the pathway and upstream regulator analyses. Virus replication was predicted to be activated, as was estrogen secretion. Previously, estrogen was shown to protect mice against severe influenza by reducing inflammation^24^ and reducing recruitment of inflammatory cells to the respiratory tract^26^. These observations are consistent with our findings showing a relationship between estrogen signaling and reduced inflammatory signaling and immune cell migration, that allow for increased viral replication and shedding even in subjects with mild disease such as those in this study.

## Discussion

We have presented a detailed exploration of early time prognosis of shedding using gene expression data obtained from human clinical challenges. The feature selection analysis indicates a strong time-dependence of the optimal discriminatory features, e.g., features that are discriminatory on the first 8 h may not be useful in day 2. We provide visual evidence that the shedders and non-shedders can be discriminated in multiple scenarios. First, the $\alpha$-$\beta$ interferon pathway, a functional pathway known to be induced in response to viral infection, clearly shows the differences in the temporal evolution of the shedders versus non-shedders. The temporal evolution of the pathway creates a biological signature that carves out a nonlinear trajectory in gene-space that undergoes an excursion that approximately returns to normal overs the course of infection. This excursion corresponds to the host immune response for shedders, while non-shedders have trajectories that remain in the general vicinity of the health state. A nonlinear visualization of this pathway illustrates a rapid departure from the health state in the first 8 h. In the machine learning experiments the features identified from retrospective studies are effectively exploited to build models of prognosis that lead to accurate predictions of shedding on prospective data in the first 32 h. The best model achieves approximately 95% accuracy in the first 8 h and 90–95% over the first 32 h. In addition, we have shown we are able to distinguish pre-symptomatic shedders from controls in the first 32 h after infection based on pooled features with over 80% accuracy. We found that the NPV predictions of our approach are especially strong and further exploration is required to understand how to improve PPVs.

The machine learning models trained on 8 h intervals and subsequent feature analysis allowed us to demonstrate the temporal evolution of the sets of the most discriminating features. This approach has the potential to elucidate the time-evolving biological processes more quantitatively; we have only attempted a preliminary exploration of this. The pooled analysis suggests an important conclusion, namely, that the best classification models will exploit time dependence.

The scope of the analysis in this paper is restricted to the study of pre-symptomatic shedders and controls in the first 32 h. This data selection allowed us to address the important question concerning prognosis at the earliest states of infection, even before the subjects reported feeling ill. Further, we were able to establish that the data indeed has predictive signatures in contrast to preceding analyses, all of which failed to predict shedding in the first 24 h. As described in^27^, 15 different models failed to produce predictions in the viral shedding challenge better than random, possibly due to overfitting. We feel the success our predictive models in this investigation is a consequence of a focus on feature selection using a carefully implemented SSVM with iterative feature removal, followed by a novel classification tool that outperforms several widely used methods. We have also used validation data to guide our parameter selection and sequestered test data during the feature selection process in order to minimize problems associated with overfitting.

A biological analysis of the feature sets used in the machine learning experiments were seen to be significant showing estrogen signaling and reduced inflammatory signaling and immune cell migration in shedders. The early suppression of inflammatory responses suggests that shedding may be associated with delayed antiviral host responses that allow the infection to become established rather than rapidly cleared by host defenses, leading to productive replication and shedding of infectious virions. The link to estrogen signaling is intriguing, particularly since sex-biased features of clinical influenza disease has been directly linked to estrogen and progesterone^22–26^. Further studies should investigate the role of sex hormones in determining the extent of shedding and if sex is a predictive factor for transmission.

While it is true that influenza A virus, RSV, and HRV are all taxonomically, genomically, structurally, and functionally distinct. They have unique tropism, use different receptors for entry, and have different mechanisms for viral genome replication, interferon antagonism, virion assembly, and egress. They also can induce virus-specific global host response profiles and result in differentially severe clinical disease and epidemiologic features. However, despite their many differences, these viruses do have many shared features, as well. They primarily infect and cause disease in the respiratory tract, are transmitted by the same routes, and notably cause asymptomatic or presymptomatic shedding in some infected persons. Although many different viral families have evolved singular approaches for replicating in the respiratory tract, these often involve common host machinery and elicit identical host response profiles, even in genetically diverse populations that are often highly variable, such as humans. Therefore, while they can be clearly extricated based on their differences, they can also be studied based on their similarities. In this case, we have focused on identifying common markers of shedding that apply across diverse respiratory pathogens in human hosts. This has substantial utility for diagnosing respiratory virus infections or predicting viral shedding based on these shared responses, where often the specific virus causing the infection is not known or cannot be rapidly determined.

This investigation ignores the possibility of co-infections. Delayed antiviral responses do likely allow many different types of viruses to establish a productive infection, leading to downstream shedding, and in some cases, disease. In this scenario, it would be possible for multiple viruses to establish a co-infection and for shedding of several different viruses. Indeed, this is thought to be the setting in which influenza reassortment occurs, which results in the shedding of reassortant progeny. We were not able to investigate this here, however, as the data used did not include co-infected individuals.

We have included four complementary machine learning tools including support vector machines (SVMs), sparse SVMs, artificial neural network (ANNs) classifiers and the supervised data reduction algorithm Centroid-Encoder. SVMs have the advantage that they are convex optimization problems and hence are fast and produce solutions that are globally optimal. The sparse penalty for SVM allows one to select features in the challenging setting where the number of variables (20000) is significantly larger than the number of data points *without an ad hoc parameter*. SVM is then applied using the resulting reduced feature set; note that in the absence of this feature reduction the problem would be mathematically under-determined and there would be an infinity of meaningless solutions. Centroid-Encoder simultaneously provides a mechanism for supervised visualization and the top performing classifier across a diverse array of methods. ANNs are used as a benchmark to compare the results of SVMs and CEs; they are widely used and provide a useful benchmarking tool. Lastly, we note that SVMs in general work well with relatively small data sets, e.g., 50–100 samples, while ANNs are viewed as data hungry. The fact that the nonlinear methods performed well, without global minima, provides strong evidence that there is enough data with enough signal to build neural network models in this investigation. These approaches may be used together as a general tool to interrogate the data for patterns and signatures related to different biological questions of interest beyond what has been explored here, e.g., shedding versus non-shedding in symptomatic individuals.

Finally, the experiments in this investigation could benefit from more samples. There have recently been additional sequencing studies on human clinical blood samples related to the host response to infection by respiratory viruses that will serve to enhance and validate the work presented here.

## Methods

### Experiments

In all experiments we employed leave one subject out (LOSO) cross validation and repeated the trials 15 times using both Subject ID and Study ID.

#### Experiment workflow

The workflow of our machine learning experiments follows these steps:

1. data partitioning
2. data normalization
3. feature selection using iterative feature removal, a technique based on sparse support vector machines
4. classification on retrospective and prospective data using a SVM, ANN and CE classifiers
5. evaluation of classification accuracy using balanced success rate (BSR)
6. functional analysis using ingenuity pathway analysis (IPA)

### Data partitioning

Here we summarize how the data was partitioned for the various experiments. We computed features on each of the four time bins in the first 32 h using to produce

- Feature Set 1: IFR applied to four influenza data sets including all H1N1 and H3N2 samples. The feature counts were 136, 38, 200 and 60 for the time-bins normalized with subject ID while the feature counts were 300, 97 500 and 200 for the time-bins normalized by study ID.
- Feature Set 2: IFR applied to H1N1 (both sets), H3N2/DEE5, HRV (both sets), and RSV. All the feature counts for Feature Set 2 were of size 200 with the exception of the time bin 25–32 h which had 130 features for subject ID normalization and 145 features for study ID normalization.
- Feature Set 3: IFR applied to H1N1 (both sets), H3N2/DEE5, and HRV (both sets). All the feature counts for Feature Set 3 were of size 200.

Each feature set has subsets associated with the time bins. We see that many of the bins were determined by an arbitrary capping of the number of features when there was no clear cut off. This ad hoc approach did not seem to significantly impact the results.

### Normalization

All microarray data is normalized at the beginning using a typical RMA normalization method^12^. The data utilized is incorporated from multiple studies and so batch effects are inevitable due to a variety of factors, including study location, the year and time of year of experiments, and procedures for processing samples for their expression values, to name a few. We employ a simple linear normalization process using either Study ID, or Subject ID and the well-known Limma process^13^. Additional details related to normalization are in the Supplementary Materials 1.

### Feature selection

Each sample has approximately 22,000 microarray probe set identification components, or features. Using entire feature sets tends to induce overfitting and poor generalization, so our work emphasizes data reduction through optimal feature selection. Our philosophy is rooted in the idea of extracting *all discriminatory features* and so we use the iterative feature removal (IFR) procedure developed in^14^.

The first step, in the feature selection process, is to identify the classes that we are proposing to discriminate. In this paper we limit the scope of the machine learning to the pre-symptomatic shedders versus pre-infection samples, or controls. Further, we partition the data by 8 h time windows. So, an example of a machine learning experiment in this paper considers controls versus shedders in time bin 1–8 h. Given these two classes, the next step is to identify a minimal set of discriminatory features using a sparse support vector classifier on a training data set informed by the balanced success rate accuracy (BSR) on a validation set. These features are then removed and a new sparse classifier is trained, again observing the error on the validation set as a stopping criterion. This process is repeated until the BSR on the validation set falls below a tolerance, e.g, $75 \%$. At this point we have a collection of all the discriminatory features associated with the data for the given data partitioning. The process is then repeated on 30 trials with different partitions. We record the frequency with which each feature is selected over the 30 trials with the idea that the significance of a feature is related to this frequency. A top fraction of the most frequently occurring features are then reordered based on the absolute magnitude of the weights. Now we create classifiers based on the resulting feature set. The accuracy plots for the ranked feature sets are presented in the Supplementary material 1.

As part of the IFR feature selection process, we use a stratified-4-fold cross validation, with test balanced success rate cutoff set to 0.5 with 50 repetitions and limit the number of iterations of IFR to 70. In any given model we retain the features selected until their is a weight ratio that exceeds 5 or a weight magnitude drops below 1e−6. For each data partition we cap the number of features that can be selected at 80% of the size of the training data. Additional details are described in the Supplementary Materials 1.

### Classifiers

Once a collection of features has been selected using sparse support vector machine based iterative feature, we build classifiers using linear support vector machines, Artificial Neural Networks and Centroid-Encoder. We describe each of these methods briefly below and direct the reader to the references for further details.

1. Linear^28–30^ and Sparse^14,31–33^ support vector machines (SVMs). In this paper we use linear SVMs for building classification models based on the features determined by sparse SVM (SSVM) Iterative Feature Removal^14^. The classical linear SVM objective function is
  1 $$\begin{aligned} \min _{\mathbf {x},b} ||\mathbf {w}||_2 + C \sum _{i=1}^n \xi _i \end{aligned}$$
  The sparse support vector machine (SSVM) is obtained by replacing the $\ell _2$-norm with the $\ell _1$-norm:
  2 $$\begin{aligned} \min _{\mathbf {x},b} ||\mathbf {w}||_1 + C \sum _{i=1}^n \xi _i \end{aligned}$$
  subject to the same set of constraints. The sparsity promoting $\ell _1$ formulation (2) can be expressed as a linear programming problem, which in turn can be solved using any number of standard algorithms; we apply a primal-dual interior point method^34^. Note that SVMs in various forms have a long history related to the analysis of gene expression data^28,30,35^.
2. Artificial Neural Networks: We apply a standard feed-forward neural network trained with one-hot-encoding to learn the labels of the training data . In all the classification tasks, we used two hidden layers with 200 ReLU activation in each layer. We used the whole training set to calculate the gradient of the error function (cross-entropy) while updating the network parameters using Scaled Conjugate Gradient Descent(SCG) see^36^ during error backpropagation.
3. Centroid-Encoder. This is a variation of an autoencoder which can be used for both visualization and classification purposes. Consider a data set with *N* samples and *M* classes. The classes denoted $C_j, j = 1, \dots , M$ where the indices of the data associated with class $C_j$ are denoted $I_j$. We define centroid of each class as $c_j=\frac{1}{|C_j|}\sum _{i \in I_j} x^i$ where $|C_j|$ is the cardinality of class $C_j$. Unlike autoencoder, which maps each point $x^i$ to itself, Centroid-Encoder will map each point $x^i$ to its class centroid $c_j$ by minimizing the following cost function over the parameter set $\theta$:
  3 $$\begin{aligned} \begin{aligned} \mathcal {L}_{ce}(\theta )=\frac{1}{2N}\sum ^M_{j=1} \sum _{i \in I_j}\Vert c_j-f(x^i; \theta ))\Vert ^2_2 \end{aligned} \end{aligned}$$
  The mapping *f* is composed of a dimension reducing mapping *g* (encoder) followed by a dimension increasing reconstruction mapping *h* (decoder). The output of the encoder is used as a supervised visualization tool, and attaching another layer to map to the one-hot encoded labels and further training by fine-tuning provides a classifier. For further details, see^19^. We use SCG^36^ to update the network parameters during error backpropagation.

### Functional analysis

Normalized array data was used to calculate expression of classifier genes from shedders relative to control samples. Log2 expression ratios were uploaded to Ingenuity Pathways Analysis (QIAGEN Bioinformatics) and analyzed using the IPA Core Analysis function. No expression or significance thresholds were applied to the classifier genes.

## Supplementary Information

Supplementary Information 1.
