# Supplementary material for: Early prognosis of respiratory virus shedding in humans
Source: Sci Rep. 2021 Aug 25;11:17193. doi: 10.1038/s41598-021-95293-z (PMC8387366; doi:10.1038/s41598-021-95293-z)
Supplement: Supplementary file 1 — Supplementary Information 1. [file 41598_2021_95293_MOESM1_ESM.pdf]

Supplementary Material: Early prognosis of respiratory virus shedding in humans

M. Aminian[3], T.Ghosh[2], A. Peterson[1], A.L.Rasmussen[4,5], S. Stiverson[1], K. Sharma[2], and M. Kirby[1,2,*]

[1]Dept. of Mathematics, Colorado State University, Fort Collins, CO 80524
[2]Dept. of Computer Science, Colorado State University, Fort Collins, CO 80524
[3]Department of Mathematics and Statistics, California State Polytechnic University, Pomona, CA
[4]Vaccine and Infectious Disease Organization-International Vaccine Centre (VIDO-InterVac), University of Saskatchewan, Saskatoon, SK, Canada
[5]Center for Global Health Science and Security, Georgetown University Medical Center, Washington, DC
[*]Corresponding author: Michael.Kirby@Colostate.Edu

May 18, 2021

1 Background Feature Selection

The biological data sets explored in this paper are characterized by the fact that the number of features, n, in each observation greatly exceeds the number of samples, p. This has been referred to as the $p \ll n$ problem [1]. Typically in machine learning models it is assumed that the number of datapoints used to build the model is much larger than the number of internal hyperparameters in the model, otherwise one runs the risk of "overfitting", essentially "memorizing" the training set and failing to have predictive power on new data. In the case of RNA transcriptomics, the number of biomarkers is on the order of tens of thousands, while the most focused classification tasks may have tens or hundreds of datapoints at most. This poses a fundamental challenge for multivariate approaches. With the considerations in the past few paragraphs in mind, much of our work is focused on applying techniques from sparse optimization to first learn small collections of discriminatory biomarkers aimed towards analyzing the time of exposure, then study the performance of classifiers on test data which focus only on these collections of biomarkers.

2 Supplementary Methods

In this section we elaborate on the procedures used towards developing predictions of viral shedding as described in the main text. From a high level view, these are broken down into feature selection, multiclass classification techniques, and analysis of results.

2.1 Balanced Success Rate (BSR)

As typical with machine learning algorithms, our goal is to build models that classify an unlabeled observation with maximum accuracy. Due to the nature of this type of experimental data, the number of members in one class versus another can be imbalanced, i.e., one of the classes may have many more samples than the other. As a simple example, suppose the task is to label 100 individuals as "healthy" or "infected" out of a collection of 90 healthy and 10 infected individuals. In this task, if the goal is to maximize simple accuracy, a naive classifier would be to classify everyone as healthy, which would result in a 90% classification rate. This is clearly not useful, as the rate of success of classification on the sick group is 0%. Various metrics can be used to account for this. For this reason we use a balanced success rate. The *balanced success rate* (BSR) for a binary classifier is the average of the true positive rate (TPR) and the true negative rate (TNR). Like ROC curves this metric accounts for minority sized classes, but has the advantage of having a direct interpretation. In the previous example, this would be an average of the 100% true positive rate, and 0% false positive rate, resulting in a 50% BSR, which is reflective of the approach used. For a binary classification, a BSR of 50% is representative of random chance. Similarly the BSR value reflective of random classification with n-classes is $100/n$%.

2.2 Normalization

The data utilized is incorporated from multiple studies and so batch effects naturally arise. All samples reveal clustering corresponding to the study in the first two principal components, indicating the need for cross-study normalization. We opt to use a stadard linear normalization process LIMMA (Linear Models for Microarray and RNA-Seq Data) [2]. Given the collection of data with labels of the study they belong, we normalize by Subject ID and StudyID as described below.

2.2.1 Linear normalization algorithm

Here we describe the mathematical detail involved in the primary normalization tool we use. Let $x \in \mathbb{R}^d$ be a single datapoint represented as a row vector, $X \in \mathbb{R}^{n \times d}$ be the data matrix. Let b be a vector whose entries are labels corresponding to the batch, with n_b total batches. The general steps are as follows. First, use one-hot encoding to map the categorical batch labels to 0-1 vectors $\{0,1\}^{n_b-1}$, where every batch i maps to a cardinal vector e_i and one batch is mapped to the origin. Then, define B to be the matrix whose rows correspond to the one-hot-encoded batch labels for each b_i. For example, if there are three unique batches and the batch labels are `b1`, `b2`, `b3`, then they are one-hot encoded to the zero vector, e_1,

and e_2. Then for a vector of batch labels (b3, b2, b1, b3), the corresponding matrix is

$$B = \begin{pmatrix} 0 & 1 \\ 1 & 0 \\ 0 & 0 \\ 0 & 1 \end{pmatrix}. \tag{1}$$

After this B is built, normalization can be done. The underlying assumption of linear batch effect removal is that the batches affect the data in a linear fashion, meaning the data matrix X can be decomposed as:

$$X = BY + \mathbf{1}^T a + X^*, \tag{2}$$

where $\mathbf{1}$ is a row vector containing the value one in all its entries. The matrix X^* is the a data matrix after accounting for a vector shift a and batch effects represented by BY. Specifically, the rows of Y represent the mean expression of the data in each batch, relative to a calculated shift a which represents the mean expression across all data.

Algorithmically, the matrix Y and vector a are chosen to minimize the residual matrix 2-norm over all possible choices $\hat{Y}$ and $\hat{a}$; a least-squares problem:

$$Y, a = \arg\min_{\hat{Y}, \hat{a}} ||X - B\hat{Y} - \mathbf{1}^T \hat{a}||_2. \tag{3}$$

To retain positivity of expression, the transformed data matrix used is

$$\widetilde{X} = X - BY. \tag{4}$$

2.2.2 Normalization using StudyID and using SubjectID

Throughout our analysis we consider two types of normalization due to "batch effects," abstractly defined. These are motivated by different, but related reasoning.

The first approach, *study normalization* is motivated by the more traditional notion of batch effects to control for heterogeneity due to environmental factors, and can be understood as studying broad trends across all subjects in the data sets as a whole. Since we are conglomerating data from eight different studies, batch effects due to study location, time, and so on, can be accounted for by applying linear batch normalization based on each sample's study label; e.g., RSV/DEE1, HRV/UVA and so on. The study labels are one-hot encoded based on the machine learning task of interest, the normalization parameters a and Y (mean expression and per-study shift) are learned and stored to transform any new data.

Exploratory data analysis shows these per-study batch effects manifest in the first two principal components of the data sets as a whole; exhibiting clustering based on study alone. This is undesirable, as any inference we may make based on this would be tainted by the knowledge that we may be "learning" features which discriminate study location, rather than underlying biology. After normalization, however, we observe this clustering to vanish.

The second approach, *subject normalization* is motivated by trying to control for heterogeneity of different individual's gene expression patterns in average value. If one treats this heterogeneity as a batch effect, the batch labels may look like RSV/DEE1 Subject 01 or HRV/DUKE Subject 22 for example. Then, all members of a batch amount to all gene expression samples for each individual for all time points of interest. The overall mean a has the same meaning, but now Y is a per-subject shift which aligns differences in overall gene expression between individuals.

2.3 Feature Selection

Feature selection techniques are of pivotal importance to avoid overfitting when dealing with omics data. Our approach is to use sparsity-promoting linear models applied to the same, or similar, classification tasks. In some sense, these are optimally generalizable, in that a model with m nonzero components must be described by m parameters (or $m + 1$, if a shift is included); see [3] for further details. Another aspect of omics datasets is strong correlation between collections of features. From the perspective of the biology, one may be interested in identifying all possible discriminatory biomarkers – as two biomarkers may strongly correlate, but one is of greater interest than the other. For this reason, we also employ an Iterative Feature Removal (IFR) scheme during the feature selection process [3]. IFR is an iterative algorithm applied to the data to extract features using sparse feature selection. The process is performed for many repetitions over different sampling of data partitions and we extract features over each data partition. This repeated application of the algorithm on different data partitions allows us to keep track of how many times each feature is extracted for different partitions and thus rank the extracted features based on frequency.

The number of genes associated to each sample is over 20,000 but in general less than 10% are useful for the characterization of shedding. Hence, the first step in our analysis is to take the subset of the top features based on frequency. Then, the feature sets are further reduced by applying the two ranking methods on the reduced set separately. In addition to frequency, we also reorder the features based on mean of absolute weights using the other output of the IFR. For each ranking method, we run a line search on the number of features, starting at one (the best feature) and going up to the end of the reduced ordered features set in increments of one. Here we train separate SVM models on the target classification problem, control vs shedders, using leave-one-subject-out cross validation for each new set of top features; see Figure 1. The criteria to select the optimal features is the number of features, under a manually selected upper limit (200 - 500 features), that produce the best test BSR. We find that the mean of absolute weights ranking method to work better than the frequency ranking alone. Finally, using these optimal features we run the control vs shedders classification using leave-one-subject-out cross validation. The experiments are performed 15 times using Linear SVM, Centeroid Encoder and Artificial Neural Network classifiers.

We extract and normalize the data for each of the four combinations and generate ranked feature set and their absolute weights using Iterative Feature Removal algorithm. The plots for the ranked feature sets are presented in Figure 2. For IFR, we use stratified-4-fold cross validation, with test BSR cutoff set to 0.5. The parameter *repetition* is set to 50. For each data partition we allow the algorithm to extract features up to max_features_per_iter_ratio = 0.8 fraction of training data samples for a maximum of *max_iters* = 70 iterations. The parameters for checking the jump in weights are set as *jump_ratio* to 5 and *jump_ratio_weight_cutoff* to 1e-6.

In this paper we apply the IFR scheme in conjunction with repeated k-fold validation to form a more robust ranking of features for the classification problems of interest.

```
Input: data, labels, num_repetition, nfolds,
       max_iters, bsr_cutoff, jump_ratio,
       jumpratio_weight_cutoff, max_features_per_iter_ratio
Output: non_zero_feature_dictionary, non_empty_abs_weights_dictionary
Initialization:
       features_dictionary ← (int, int)
         // key - feature_index, value - feature frequency.
         // Initialize all values to zero for all features
       absolute_weights_dictionary ← (int, [ ])
         // key - feature_index, value - List of weights.
         // Initialize all values to empty arrays for all features
for n_rep = 1 to num_repetition do
    Partition data and labels into train and test set using n_folds stratified split
    for partition in partitions do
        list_of_features_for_current_data_fold ← [ ]
        list_of_weights_for_curr_fold ← [ ]
        mask_array = [1] * data.shape[1]
        // Array of ones with size = number of features.  It tracks features which haven't been removed
        for i = 1 to max_iters do
            1. Instantiate a Sparse Support Vector Machine classifier
            2. Select active training and test data using Mask array
            3. Fit SSVM on active training data and labels
            4. Calculate BSR for active test data
            5. Break if BSR is below bsr_cutoff
            6. Extract ssvm weights
            7. Convert weights to absolute values and sort largest to smallest.
            8. Calculate ratio of successive sorted absolute weight values
            9. Detect jumps in the coefficient values using jump_ratio parameter.
            10. Break if sufficient jump is not found
            11. Break if the weight at the jump is smaller than jumpratio_weight_cutoff
            12. selected_feature_count = index_of_first_jump + 1
            13. Break if the number of selected features is greater than cutoff
              // cutoff = max_features_per_iter_ratio * num_train_data_samples
            14. Select features, from the sorted list, up to first jump index
            15. Set entries for the selected features in mask array to 0
            16. Append selected features to list_of_features_for_curr_fold
            17. Append absolute weights for selected features to list_of_weights_for_curr_fold
        end
        Update features_dictionary using the features selected for current fold
        // increment frequency by 1 for each selected feature
        Update absolute_weights_dictionary using features selected for current fold
        // append absolute weight for each selected feature
    end
end
Extract non_zero_feature_dictionary from features_dictionary
Extract non_empty_abs_weights_dictionary from absolute_weights_dictionary
return non_zero_feature_dictionary, non_empty_abs_weights_dictionary
```

Algorithm 1: Iterative Feature Removal with extensions.

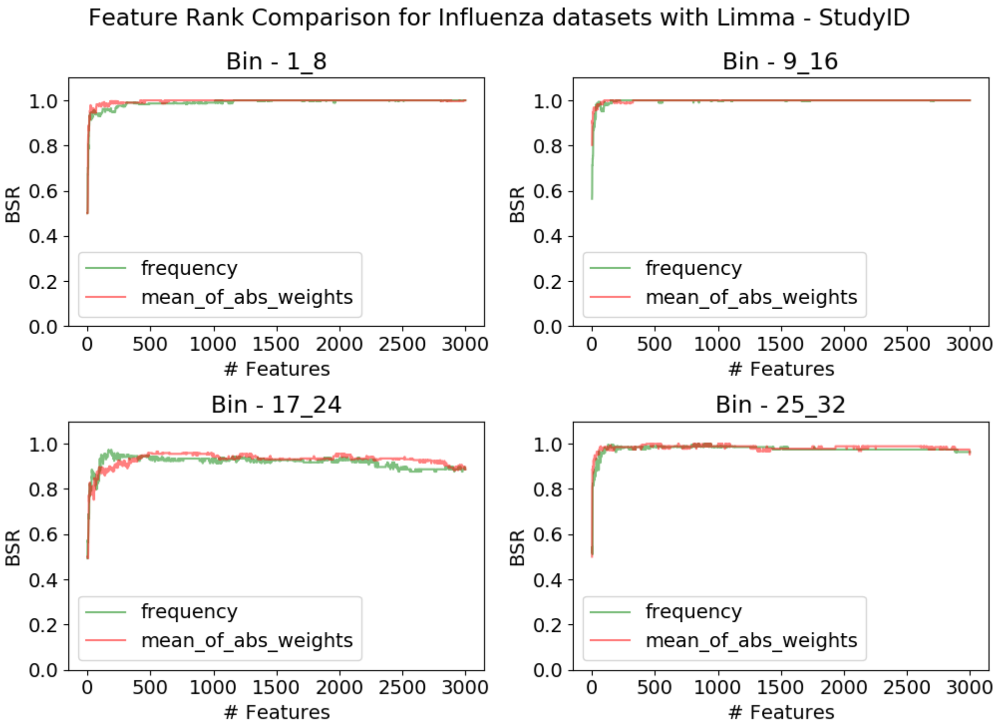

Figure 1: Feature Set 1 selection using Limma-StudyID. The classification accuracy (BSR) as a function of the number of features is plotted for each time bin.

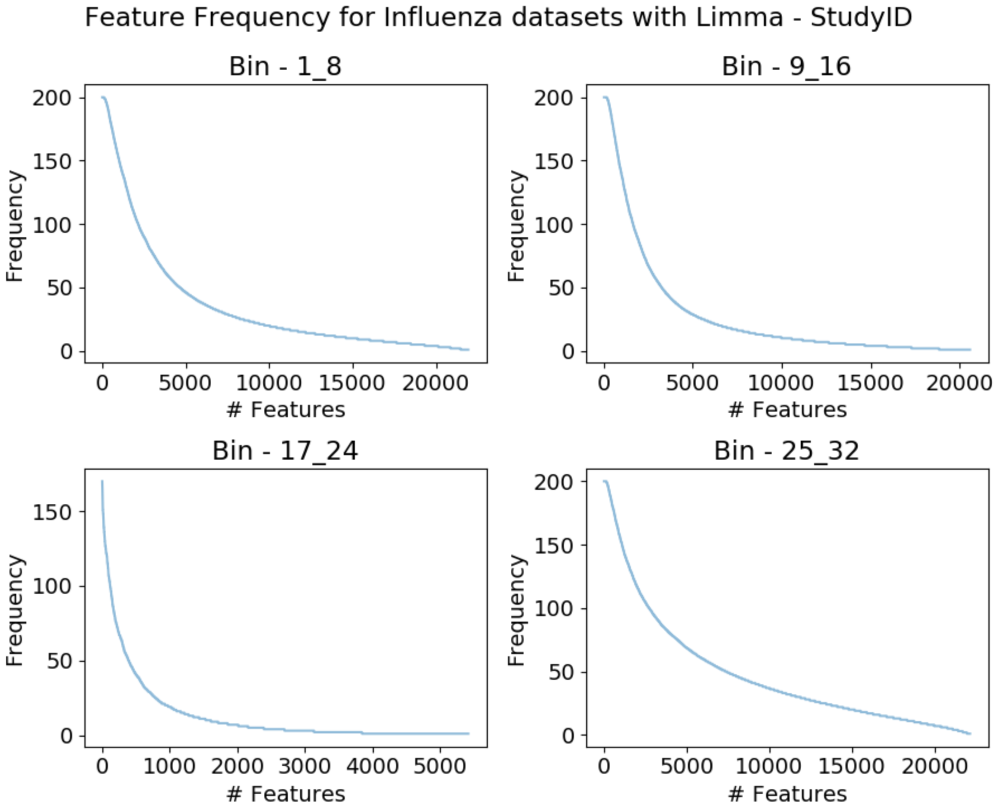

Figure 2: Feature Set 1 ordering by frequency using Limma-Study ID preprocessing.

References

[1] Hastie, T., and Tibshirani, R. Expression arrays and the $p \gg n$ problem. *See¡ http://www-stat. stanford. edu/ hastie/Papers/pgtn. pdf* (2003).

[2] Ritchie, M. E., Phipson, B., Wu, D., Hu, Y., Law, C. W., Shi, W., and Smyth, G. K. limma powers differential expression analyses for rna-sequencing and microarray studies. *Nucleic Acids Research 43*, 7 (2015).

[3] O'Hara, S., Wang, K., Slayden, R. A., Schenkel, A. R., Huber, G., O'Hern, C. S., Shattuck, M. D., and Kirby, M. Iterative feature removal yields highly discriminative pathways. *BMC genomics 14*, 1 (2013), 832.
